# Enzyme immobilization studied through molecular dynamic simulations

**DOI:** 10.3389/fbioe.2023.1200293

**Published:** 2023-06-08

**Authors:** Nicholus Bhattacharjee, Lur Alonso-Cotchico, Maria Fátima Lucas

**Affiliations:** Zymvol Biomodeling SL, Barcelona, Spain

**Keywords:** enzyme immobilization, molecular dynamics simulations, nanoparticles, self assembled monolayers, graphene, carbon nanotube

## Abstract

In recent years, simulations have been used to great advantage to understand the structural and dynamic aspects of distinct enzyme immobilization strategies, as experimental techniques have limitations in establishing their impact at the molecular level. In this review, we discuss how molecular dynamic simulations have been employed to characterize the surface phenomenon in the enzyme immobilization procedure, in an attempt to decipher its impact on the enzyme features, such as activity and stability. In particular, computational studies on the immobilization of enzymes using i) nanoparticles, ii) self-assembled monolayers, iii) graphene and carbon nanotubes, and iv) other surfaces are covered. Importantly, this thorough literature survey reveals that, while simulations have been primarily performed to rationalize the molecular aspects of the immobilization event, their use to predict adequate protocols that can control its impact on the enzyme properties is, up to date, mostly missing.

## 1 Introduction

**FIGURE 1:**
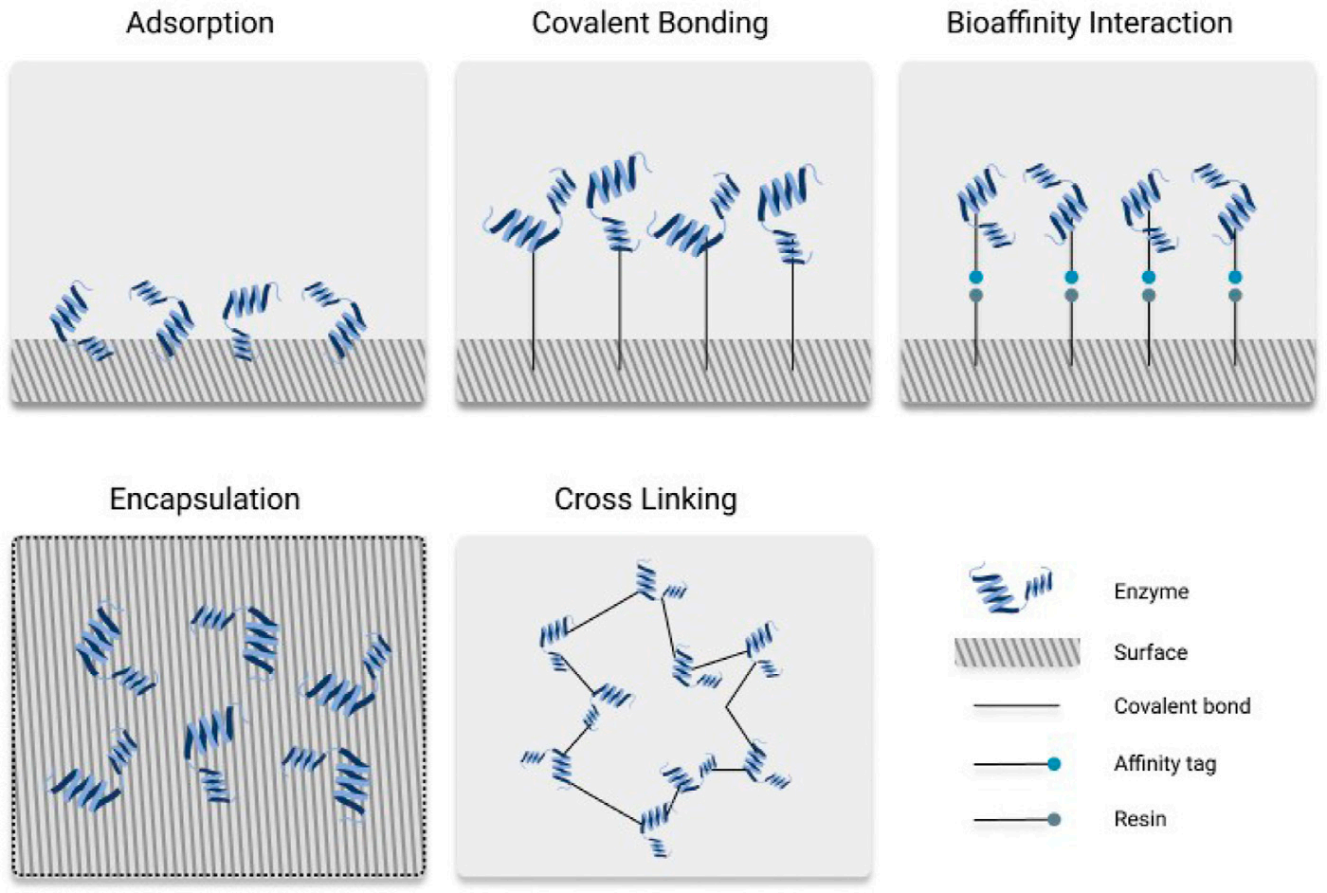
An illustration for different types of enzyme immobilization methods.

Enzymes are diverse natural catalysts able to perform a wide range of chemical reactions with high specificity and selectivity. In addition to these inherent properties, their ease of production, sustainability and often low cost compared to several metal catalysts (Sheldon, 2018; Adams et al., 2019; Heckmann and Paradisi, 2020) has been steadily increased the industrial use of enzymes (Choi et al., 2015; Singh R. et al., 2016; Chapman et al., 2018; Abdelraheem et al., 2019; Wackett, 2019; Wu et al., 2021). Due to these advantages, biocatalysis is nowadays applied in a wide variety of sectors, ranging from agrochemicals (Aleu et al., 2006) to textiles (Madhu and Chakraborty, 2017), cosmetics (Sá et al., 2017; Yarosh et al., 2019), commodity chemicals (Woodley, 2020), detergents (Al-Ghanayem and Joseph, 2020), food (Raveendran et al., 2018), leather (Khambhaty, 2020), paper and pulp (Hakala et al., 2013; Singh G. et al., 2016), biomaterials, and (bio)pharmaceutical manufacture (Meghwanshi et al., 2020). However, despite their excellent features, a full exploitation of enzymes’ industrial potential has not been yet achieved. This is due to many reasons like instability in extreme pH, high temperature, presence of surfactants, solvents, or metal ions; short shelf-life; and high substrate concentrations (Sheldon and Woodley, 2018; Silva et al., 2018; Basso and Serban, 2019; Sheldon, 2019). In the last decades, the development of enzyme engineering approaches has aided to overcome these issues while bridging the gap between biocatalysis and industry. Enzyme tuning throughout rational design, directed evolution and, more recently, by the use of *in silico* tools has been key to speed up the application of enzymes into industry. These efforts aimed to specialize the enzymes towards their target reactions have led to a wide range of examples in which properties like activity, substrate specificity, selectivity and stability have been successfully increased (Chen, 2001; Chowdhury and Maranas, 2020; Nirantar, 2021). Still, these strategies are in some cases not enough to meet the industrial needs, especially when the experimental conditions are too hazardous leading to enzyme inhibition or when the process is not efficient enough due to limitations in enzyme recovery and recycling. Enzyme immobilization has been used as one of the most prominent methods to overcome these flaws and further boost the enzyme’s application (Garcia-Galan et al., 2011; Tran and Balkus, 2011; Zdarta et al., 2018). The term “immobilized enzymes” have been defined as “enzymes physically confined or localized in a certain defined region of space with retention of their catalytic activities, and which can be used repeatedly and continuously” (Brena et al., 2013). This provides various practical advantages which include cost savings, easier reusability, enzyme recovery, generally improved enzyme stability under storage and operating conditions, enhanced activity, optimized selectivity or specificity, etc (Mateo et al., 2007a; 2007b; Barbosa et al., 2013; 2015; Secundo, 2013; Sheldon and van Pelt, 2013; Mohamad et al., 2015; Mehta et al., 2016; Nguyen and Kim, 2017; Bernal et al., 2018; Rajangam et al., 2018; Ramakrishna et al., 2018; Zdarta et al., 2018). The first immobilized enzyme was discovered more than a century ago while demonstrating that activity of an invertase enzyme is not hampered when it is adsorbed on a solid matrix, such as charcoal or an aluminum hydroxide (Nelson and Griffin, 1916). However, it was not until the end of the twentieth century that considerable interest in the combination of enzymes with materials was raised with the path breaking work by Klibanov, Russell, Halling, and others on the preparation and utilization of immobilized enzymes (Klibanov, 1979; 1983; Yang et al., 1995; Zacharis et al., 1997; LeJeune et al., 1998), including strategies such as covalent attachment, encapsulation, adsorption on solid supports, entrapment in polymeric gels, cross-linking and PEGylation. Nowadays, the enzyme immobilization has widely spread and has been used successfully in applications like antibacterial or/and antifouling coatings (Banerjee et al., 2011; Yu et al., 2011), industrial catalysis (Liese and Hilterhaus, 2013), drug delivery (Liu et al., 2009; Liese and Hilterhaus, 2013), biosensors (Rusmini et al., 2007; Talbert and Goddard, 2012), and biofuel cells (Minteer et al., 2007). Despite its potential, immobilizing enzymes is still a growing field and there is no universal method or carrier material used for this purpose. Instead, in accordance with different kinds of enzyme attachment, enzyme immobilization strategies can be classified into physical adsorption, encapsulation, entrapment, cross-linking, covalent attachments, and bioaffinity interactions (Figure 1).

Although immobilization is nowadays widely used and incorporated in standard experimental pipelines, the mechanism of immobilization has not been satisfactorily clarified at the microscopic level owing to the complexity of the immobilizing agent and the molecular nature of the protein-surface interactions. Furthermore, how the immobilization event can ultimately affect the enzymatic efficiency is a complex process which is poorly understood and difficult to rationalize. Molecular simulations can help in deciphering the mechanisms of chemical and biological processes as well as be used for design and development of new products. Hence, with access to highly developed fast computers in recent years, molecular simulation techniques have become a powerful tool to investigate the immobilization phenomenon at molecular level and can act as strategic complement to experiments. Among the available simulation techniques, Molecular Dynamics (MD) simulations, which can disclose the microscopic nature of intermolecular interactions, have been used successfully to investigate the immobilization of enzymes over the last two decades. Some recent reviews have discussed adsorption, the initial step of immobilization, using simulation techniques on various surfaces (Ganazzoli and Raffaini, 2019; Quan et al., 2019). Other reviews have highlighted the MD simulation studies of nanoparticle interactions with proteins and other biomolecules (Khan and Nandi, 2014; Brancolini and Tozzini, 2019; Casalini et al., 2019), or the protein corona, which forms protein layers on the nanoparticle surface (Lee, 2021). However, a detailed description on the use of *in silico* methods to assess the structural aspects of the enzyme immobilization is lacking.

In this review, we discuss how MD simulations -either all atoms or coarse grained- have been employed to understand the surface phenomenon in the enzyme immobilization procedure, in an attempt to decipher its impact on the enzyme activity. These are presented in this article based upon different types of surfaces on which the immobilization of the enzymes was carried out. The subsequent part of the review begins with discussion of MD simulations performed to study enzyme immobilization on different types of nanoparticles followed by similar works done on Self Assembled Monolayers (SAMs). Works involving graphene or carbon nanotubes are presented thereafter. This is followed by discussion on other surfaces used for enzyme immobilization and have been rationalized by MD simulations. We also briefly discuss surfaces used for immobilization for which simulation studies are lacking, emphasizing possible difficulties in studying such surfaces. These include polymers, metal-organic-framework (MOF) or metal based affinity tags. A thorough literature survey performed for writing this review revealed that, while the distinct studies have been focused on rationalizing the molecular aspects of the immobilization process, the use of computation to predict adequate protocols that can control the impact on the enzyme properties is very limited. The final section of this article discusses the few recent proposals for predicting immobilization protocols using MD simulations and future perspectives in this direction.

## 2 Immobilization on nanoparticles

**FIGURE 2:**
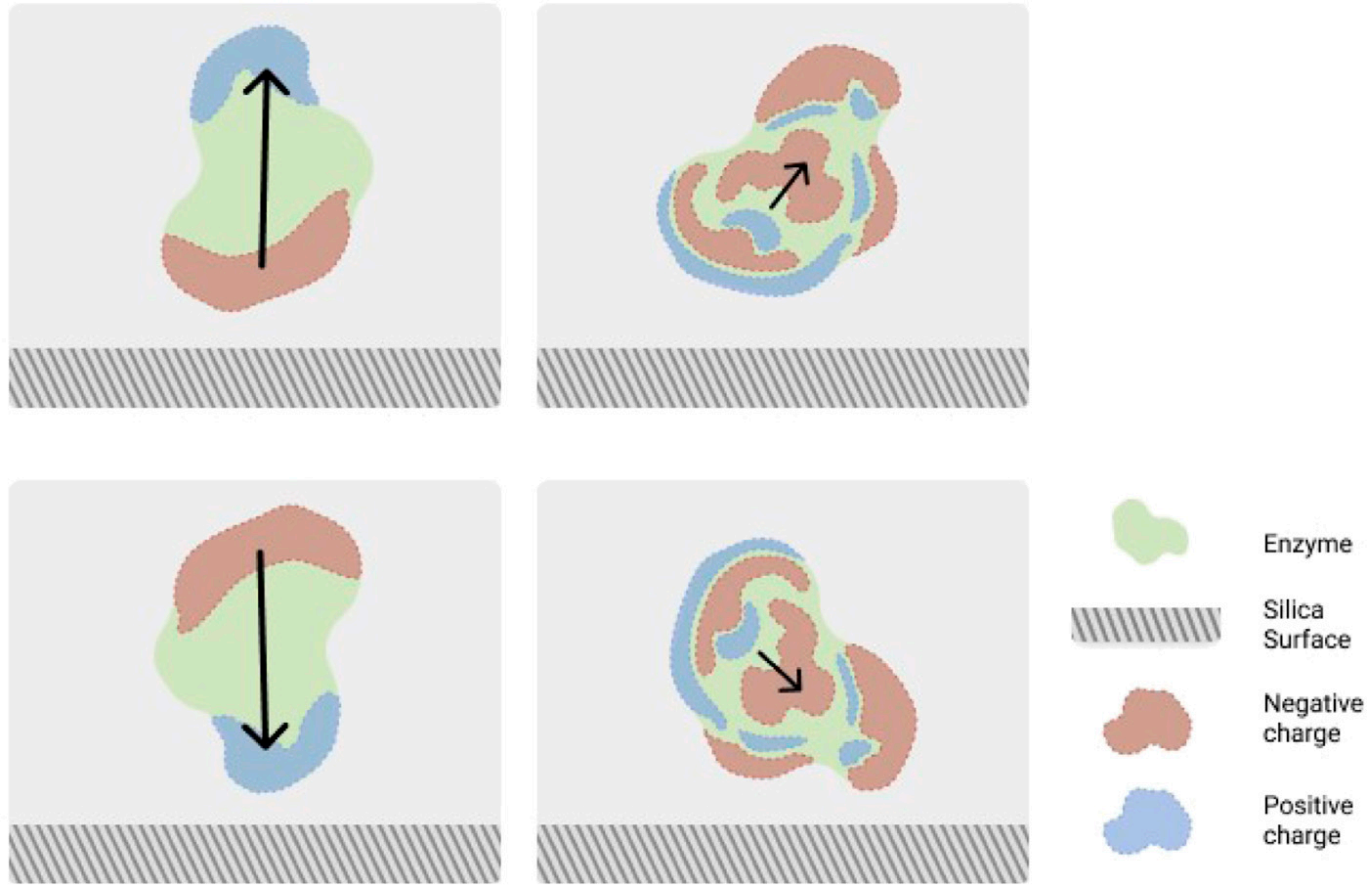
Schematic view of most (bottom) and least (top) attractive orientations of chymotrypsin (left) and lysozyme (right) placed over the SiO_2_ surface. The scheme is based upon Figure 4 from Hildebrand et al., 2015.

The immobilization of enzymes using nanoparticles offers some advantages such as: high enzyme loading, improved enzyme stability, and ease of separation from the reaction products (Cao et al., 2003). Among these, the surface of silica nanoparticles (SNPs) is often used for enzyme immobilization due to the high adsorption capacity of the nanoporous silica particles. One example is the study of the papain enzyme and its adsorption mechanism, that was studied by classical MD simulations (He et al., 2014). The results showed that papain, following initial binding to silica, optimizes its structure which allows more of its atoms to come in contact with the surface reaching the most favored immobilization mode. Although the major secondary structures were preserved, small rearrangements of structures at the entrance of the catalytic site were observed. These led to an increase in the accessibility of the active site for the solvent as well as the substrates, which could therefore facilitate productive binding modes between the substrates and the enzyme. In the same year, Sun et al. used MD simulations to study the orientation and adsorption of three different enzymes, namely, cytochrome c, RNase A and lysozyme on SNPs (Sun et al., 2014b). These enzymes were found to be induced with greater structural stabilization by small SNPs and the results indicated selective interactions between the enzymes and SNPs, where deprotonated silanol groups were used leading to silica with a negative surface charge. Hildebrand et al. (2015) also showed, through the use of MD simulation, that ***α***-chymotrypsin and lysozyme, both positively charged, have preferential binding modes to the amorphous silica based on their surface residues. They showed that ***α***-chymotrypsin, with its ***α***-helical domain turned towards the surface, has a preferred adsorption orientation while lysozyme shows not so clear preference in the orientation. This is due to the fact that ***α***-chymotrypsin has a large dipole moment, leading to preferential adsorption through its positive surface-potential region, while its negative surface-potential region is exposed toward the solvent. However, lysozyme has more homogeneously distributed surface-potential and hence much less pronounced orientational preference. A schematic view of this is shown in Figure 2. In a later work Yu and Zhou. (2016) used coarse grain MD simulation to study the adsorption of lysozyme, which showed a narrow orientation distribution on the silica surface. Interestingly, with increasing nanoparticles size greater conformational changes were observed. This indicates that a decrease in the nanoparticle’s surface curvature may result in a higher degree of electrostatic interactions with the enzyme which can perturb the dynamics of the enzyme. These effects are not due to the area of contact between lysozyme and SNPs. Instead, they are because of the dissimilarities in the interfacial layer of hydration found over the SNPs with different sizes. This is due to the fact that strength of interfacial hydration is inversely proportional to the nanoparticle curvature. Hence, a more ordered distribution of interfacial water molecules is observed. In addition to that, negatively charged SNPs are found to have less effect on the conformation of lysozyme. This phenomenon is more pronounced for larger SNPs at greater ionic strength. In a more recent work, coarse grain MD and constant-pH Monte Carlo (MC) simulations were used to study the lysozyme adsorption on negatively charged SNPs. The results showed that the increase of pH leads to changes in orientation of the adsorbed lysozyme when the solution pH gets closer to the enzyme’s isoelectric point (Caetano et al., 2021). MD simulations were also used by Wang et al. (2020) to tune the immobilization of a lipase on SNPs. Along with structural analysis and catalytic characterizations the results confirmed that the reoriented lipase immobilized through hydrophobic adsorption in its open conformation was crucial for achieving the highest efficiency in the catalytic process.

Other types of nanoparticles, such as gold-, silver- or titanium-based have been used for enzyme immobilization. All atom MD simulations in combination with absorption, fluorescence and infrared spectroscopy were used to investigate the interaction between silver nanoparticles (AgNPs) and a catechol O-methyltransferase (Usman et al., 2021). The results suggested that AgNPs influence the catalytic activity of the catechol O-methyltransferase by interacting with six amino acids from four of the enzyme’s predominant helical structures which are in close proximity to the active site, and therefore own the potential to control the enzyme features. Baig et al. (2015) studied the immobilization of a yeast alcohol dehydrogenase on polypyrrole–titanium(IV)phosphate (PPy–TiP) nanocomposite. The hydrogen bonding and van der Waals interactions were observed to play an important role in the formation of complexes which are favored energetically. The estimated activity of the enzyme was consistent with the experimental findings. More recently, Tavanti et al. (2019) studied the adsorption of trypsin along with myoglobin and hemoglobin over gold (Au) nanoparticles (AuNPs) using coarse grained MD simulations. No significant conformational changes were observed in this case and the AuNP binding site did not interfere with important functional sites of the enzyme. AuNPs were also prevalently functionalized with organic thiol molecules prior immobilization leading to a self assembled monolayer, which will be discussed in the next section. Although out of the scope of the current review, in the last years, these computational studies have not been limited only to the study of enzymes, but they also have been applied to assess the binding of antibodies, nanobodies and others with nanoparticle surfaces (Xiao et al., 2018; Simões et al., 2021; Martí et al., 2022).

## 3 Immobilization on self assembled monolayers (SAMs)

**FIGURE 3:**
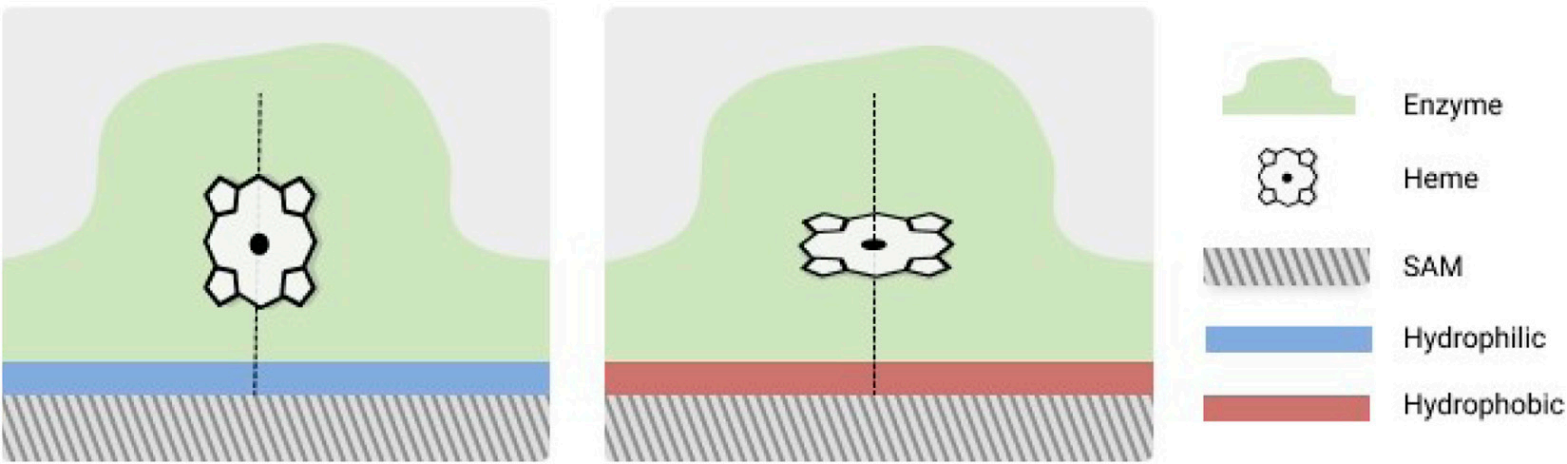
Diagrammatic representation of cytochrome c heme group orientation with respect to different SAM surfaces. The scheme is based upon Figure 2 from Zhou et al., 2004, Figure 12 from Xie et al., 2015, Figure 3 from Rivas et al., 2005 and Figure 6 from Peng et al., 2016.

Self assembled monolayers (SAMs) are formed by a pool of thiol molecules located over gold surfaces in an organized manner. SAMs offer a perfect platform for studying immobilization of enzymes due to their adjustable structures and ease of functionalization. These properties allow SAMs not only to be used as immobilizing material but also as membrane mimics to better understand the nature of the protein-membrane interactions (Ulman, 1996; Smith et al., 2003; Yeung et al., 2018). One of the earliest *in silico* studies of a protein interacting with a SAM was performed by Tobias et al. (1996). They investigated the behavior of cytochrome (cyt) c covalently attached with hydrophilic (SH-terminated) and hydrophobic (CH_3_-terminated) SAMs. The results suggested that the enzyme undergoes minor structural changes when attached to these surfaces. However, the changes are still significant with the enzyme being less spherical when attached to the hydrophilic SAM with the polar surface residues reaching out for making interactions with the SAM surface. Regarding the orientation of the enzyme, it is such that the heme plane is almost parallel to the surface when attached to the hydrophobic SAM, whereas it is nearer to the perpendicular orientation when attached to the hydrophilic surface (Figure 3). Subsequent studies have further investigated the nature of the interactions between cyt c and different types of SAMs (Nordgren et al., 2002; Zhou et al., 2004; Rivas et al., 2005; Xie et al., 2015; Peng et al., 2016). Nordgren et al. (2002) further studied the effect of SAMs in the enzyme properties by introducing several modifications such as tuning the polarity of the SAM end groups, the degree of hydration around the monolayer and the coordination number of the heme iron present on the cyt c. The overall structure of the enzyme was found to be preserved, while the SAM structure was perturbed only in regions with direct contact to the enzyme. The work of Zhou et al. (2004) have shed more light on the orientation of cyt c with respect to negatively charged carboxyl-terminated SAM. They have shown that the most efficient orientation of the enzyme places the heme group perpendicular to the SAM surface. In combination with spectroscopic experiments Rivas et al. (2005) performed MD simulations of cyt c docked into negatively charged SAM via its lysine rich domain. The results from the work suggested that cyt c, upon binding to biomembranes or partner proteins, may have electric-field-induced redox potential shift and hence may affect the biological electron transfer process of cyt c. Xie et al. (2015) performed MD simulations to further probe the modulation of cyt c behaviors on the zwitterionic phosphorylcholine SAMs which are electrically responsive. The results showed that it is possible to regulate enzyme behavior through observing deformation of the enzyme and application of electric fields on responsive surfaces, The behaviors regulated were promotion or retardation of enzyme adsorption and regulation of enzyme orientation. Peng et al. (2016) studied the phosphate and chloride ions effect on the adsorption of cyt c using MD simulations. The results showed unstable adsorption of cyt c on the surface in presence of chloride ions despite relatively high ionic strength. Alternatively, the presence of phosphate ions was found to promote stable adsorption.

**FIGURE 4:**
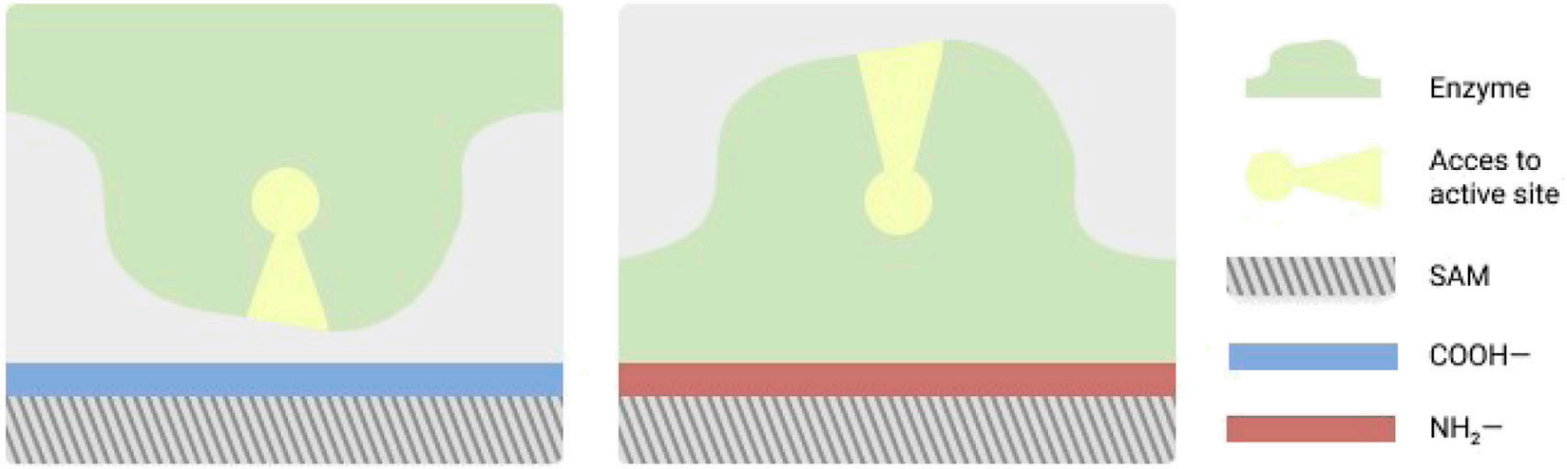
Scheme showing orientation of the RNase A active site with respect to different types of SAM surfaces. The scheme is based upon Figure 3 from Liu et al., 2015b.

In addition to cyt c, the Zhou group have studied adsorption behaviors of other enzymes upon immobilization on SAMs using a protocol which combines both MC and MD simulations (Xie et al., 2013; 2020; Liu et al., 2015a; Liu et al., 2015b; Zhao et al., 2015; Liu et al., 2017; Yang et al., 2018; Yang et al., 2020). The protocol involves first a quick sampling of the protein over the surface using MC for predicting which is the dominant orientation of the enzyme. Then, MD simulation is used to obtain atomic level adsorption details such as contacting residues, conformational changes, interaction energy, etc. Using this protocol, the adsorption of lysozyme on carboxylated SAMs under the effect of an external electric field was studied by Xie et al. (2013). It was observed that lysozyme is adsorbed with “bottom end-on” or “side-on” orientation in absence of electric fields. However, when an electric field is applied, it preferes “side-on”, “back-on” and “top end-on” orientations. This supported the possibility of tuning the immobilization process of a protein with the desired orientation by applying different electric fields. Zheng et al. (2004), Zheng et al. (2005) studied the interaction of lysozyme with alkanethiolate SAMs terminating with different chemical and observed that the flexibility and the conformation of the SAMs together with bound water near the interface are features responsible for strengthening the interaction between the enzyme and the SAM. A similar observation was made by He et al. (2008) while studying the nature of the lysozyme interactions with zwitterionic phosphorylcholine-terminated SAMs. In a more recent work, Xie et al., 2020 studied the lysozyme adsorption on electrically responsive carboxyl/hydroxyl SAM, further supporting that the behavior of the lysozyme over the SAM surface is affected by the nature of the applied electric field to the system. The combined MC and MD protocol was also used by Liu et al. (2015a), Liu et al. (2015b), Liu et al. (2017) to study the conformation and orientation of a feruloyl esterase, laccase and ribonuclease A (RNase A) adsorbed on SAMs. Two different types of SAMs-positively charged NH_2_−SAM and negatively charged COOH−SAM-were used for these studies. RNase A was found to adsorb on both the charged SAMs in opposite orientations, i.e., when adsorbed on COOH−SAM RNase A it was oriented towards the surface, while on NH_2_−SAM, the active site was oriented towards the solution (Liu et al., 2015b). Figure 4 schematically shows this active site orientation with respect to different types of SAMs. Based on these studies, they concluded that the adsorption over negatively charged surfaces could be used to remove the redundant RNase A, while the adsorption over positively charged surfaces favors the enzymatic activity of RNase A. This control of the orientation by using different charged surfaces was also observed for feruloyl esterase from *Aspergillus niger* (AnFaeA) (Liu et al., 2015a). In addition, ionic strength (IS) and surface charge density (SCD) effects were also considered and the results suggested that positively charged surfaces at high IS and low SCD can maximize the immobilized AnFaeA utilization. In a later work by Liu et al. (2017), it was observed that for a laccase the orientation of the electrodes is very important for achieving a fast direct electron transfer (DET)-and hence to find a small pathway-between the substrate and the T1 copper site during the immobilization of laccase immobilization. They studied the *Trametes versicolor* laccase (TvL) immobilized on COOH−SAM and NH_2_−SAM, showing that the T1 copper site of TvL is closer to the positively charged surface. This is due to the TvL orientation on a negatively charged surface being broader in comparison to its orientation on a positively charged surface, which leads to more conductivity in the latter’s case. In contrast, a similar work using bilirubin oxidase showed that negatively charged surfaces were more favorable for the direct electron transfer (Yang et al., 2018). In another separate work, it was shown that the enhanced catalytic activity of a laccase immobilized in SAM was ascribed to high hydrophobic interaction energy (Miyazawa et al., 2017). The combined MC and MD protocol was also used to study *Candida antarctica* lipase B (CalB) adsorption on hydrophobic graphite surface and hydrophilic TiO_2_ surface, as well as on both positively and negatively charged (NH_2_- and COOH-) SAMs (Zhao et al., 2015). The results show that CalB forms strong adsorption on both hydrophobic and hydrophilic surfaces. When adsorbed to NH_2_-SAM the catalytic center of CalB is oriented towards the surface and hence not preferable for binding of the substrate. While when it is adsorbed on the COOH-SAM the catalytic center is facing the solution and is preferable for substrate binding. In a more recent work, Yang et al. (2020) have performed simulations of acetylcholinesterase from *Torpedo californica* (TcAChE) immobilized on COOH- and NH_2_- SAMs. Positively charged NH_2_-SAM surface was able to provide a better microenvironment needed for coherent bio-catalytic reaction of the enzyme. This led to quicker DET between the enzyme and the electrode surface. A more recent work emphasized the orientation and activity of enzymes while tethered into SAMs. Li et al. (2018) studied the immobilization of two variants of β-glactocidase tethered on a SAM surface terminated with pure maleimide and mixed hydrophilic surface made of hydroxyl groups and maleimide. Using coarse-grained MD simulations together with sum frequency generation (SFG) vibrational spectroscopy, they showed that the orientation of the immobilized enzyme plays an important role in its activity: A significant increase in activity was observed upon immobilization on mixed SAM in comparison to pure SAM. In further advanced work by the same group using a nitro-reductase (NfsB) as a model enzyme it was shown that two strategically placed surface tethering points could provide a better catalytic efficiency and stability (Zou et al., 2018). These immobilization sites were designed based upon the coarse-grained MD simulation results, and variants of the enzyme with cysteinyl residues at these sites were expressed and purified. These variants were thereafter immobilized upon maleimide terminated SAM. The results showed that in comparison to the enzyme tethered at a single site, immobilizing variants of NfsB using two tethering positions display general improvement in thermal stability.

Other studies have been focused on the immobilization of enzymes over SAM coated Au nanoparticles. The catalytic activity and structural integrity of an oxygen-tolerant [NiFe] hydrogenase on Au electrodes coated with SAM were studied using surface enhanced infrared adsorption (SEIRA) spectroscopy, atomic force microscopy (AFM), and protein field voltammetry (PFV) in combination with short all-atom MD simulations (Heidary et al., 2015). A more recent multiscale simulation work merging all-atom MD and coarse-grained Brownian dynamics simulation to study the adsorption of β-glucosidase A (βGA) on SAM-functionalized as well as bare gold surfaces (Bourassin et al., 2022). In this work it was observed that although there was little impact on the enzyme conformation, the adsorption process perturbed the mechanical properties and the catalytic activity of the enzyme. Although the βGA adsorption on SAM-functionalized surfaces is less stable than the bare gold, it is more specific and causes less disruption to enzymatic function.

## 4 Immobilization on graphene oxide and carbon nanotube

In the recent past, graphene and graphene oxide (GO), a graphene derivative which is soluble in water, have captivated immense attention due to their interesting chemical and physical properties. In particular, GO is found to be an ideal candidate for enzyme immobilization since it needs neither modification of the surface nor coupling reagents because it is enriched with oxygen-containing groups. Sun et al. (2014a) studied the interaction of α-chymotrypsin (ChT) with both graphene and GO. Although ChT was found to be adsorbed onto both surfaces, the hydrophobic and cationic residues of ChT interact stronger with GO, leading to the active site deformation and therefore its inhibition. In another work, Zhao et al. (2018) studied the orientation of cyt c on graphene and GO, supporting the hypothesis elucidated in the previous section which states that the orientation of cyt c on immobilizing surfaces is crucial for the electron transfer (ET). Using MD simulations, they investigated the conformational change, the pathways of ET, and dominant driving forces to understand the conformation, binding, and bioactivity of cyt c. It was observed that, in comparison to graphene, the cyt c heme plane was deviated moderately from the standard location and Met-80, the axial ligand, was more adjacent to the GO surface, facilitating the ET. Another enzyme family that has been immobilized using GO includes lipases. The immobilization of lipases, enzymes with several industrial applications, faces a key challenge related to opening the enzyme lid domain and maintaining this open conformation for its active site exposure. This can be achieved and tuned through chemical reduction of GO, which allows modulating the enzyme activity. MD simulations were used to study the molecular mechanisms driving the lid-opening, shedding light on the key role of the hydrophobic interactions occurring between the interface of the lipase and GO (Mathesh et al., 2016). Additionally, Zhuang et al. (2020) observed that the lipase immobilized on functionalized GO is around 20 times more active than when immobilized on GO. Regarding the effect of GO on enzyme stability, Li et al. (2021) showed that the thermal stability of D-psicose 3-epimerase (DPEase) enzyme is improved upon immobilization. Aiming to rationalize this effect all atom MD simulations of DPEase complexed with its natural substrate D-Fructose were performed with and without anchoring to GO. It was observed that the active site of DPEase is stabilized at high temperature in presence of GO. This is due to the fact that strong interaction between DPEase and GO can prevent the loops α1′-α1 and β4-α4 of the DPEase, which contains the active site residues, to drastically fluctuate. The simulation results supported previous experimental observations about the improvement in thermal stability of DPEase upon immobilization on GO. In contrast, the immobilization of the hen egg white lysozyme (HEWL) on GO led to active site blocking, affecting the flexibility of surrounding residues and hampering the activity (Bera et al., 2018). In a separate work, Ray et al. (2018) used GO to study the structure-function relationship of the nucleoside diphosphate kinase (NDPK), whose inhibition may be a potential therapy for patients with end-stage heart failure. MD simulations suggested that the interaction of GO with the NDPK His118 residue is very favorable, leading to the inhibition of adenosine triphosphate (ATP) binding to the enzyme, which can otherwise trigger mutated G protein phosphorylation. More recently, the interaction between COVID-19 protein from severe acute respiratory coronavirus (SARS-CoV-2), 3C-like (3CL) main protease (M^pro^), with intact graphene (IG), defective graphene (DG) and GO, was investigated (Wang et al., 2022). It was observed that DG and GO interact with M^pro^ more intensely making its overall structure to become more flexible. Furthermore, it was shown that, in contrast to IG and GO, DG can inactivate M^pro^ and inhibit its expression effectively by hampering its active pocket.

Due to their unique thermal, mechanical and biocompatible characteristics, carbon nanotubes (CNTs), among all nanomaterials, are a very promising surface for supporting enzymes (Zaboli et al., 2019). Some of the first MD simulation studies elucidating protein-CNT interactions include proteins such as the Coxsackie-Adenovirus Receptor (CAR) (Johnson et al., 2009), human serum proteins (Ge et al., 2011), lysozyme (Vaitheeswaran and Garcia, 2011). Johnson et al. used all-atom MD simulations of the CAR and the CAR-Knob complex covalently attached to CNT to assess the degree of structural deformation upon binding. It was observed that, despite significant structural fluctuations, the overall structure of CAR underwent minor deformation from its native structure and did not affect the CAR’s ability to bind Knob. These results supported that CAR retains its biological functionality when attached to CNT. Ge et al. performed all-atom MD simulations in conjugation with experimental approaches to investigate the interactions of human serum proteins with single-wall CNTs (SWCNTs), finding a binding competitiveness of these proteins with different adsorption capacities and packing modes. They observed that π-π stacking interactions between SWCNTs and the aromatic residues of the protein are critical to the adsorption capacity. This in turn affects cellular responses resulting in different degrees of cytotoxicity. Vaitheeswaran and Garcia used coarse-grained replica exchange MD simulations to study the stability of lysozyme on CNT and found that it is dependent on the equilibrium between the unfavorable enthalpy and favorable entropy change upon adsorption. Subsequent work using all-atom MD simulations showed that the lysozyme-CNT interacting region is far away from the catalytic site, leading to intact catalytic activity. It was also observed that the Aminic and Amidic moieties of the protein behave like surfactants, blocking the access of the solvent to the CNT (Calvaresi et al., 2012). In a separate study, Zhang et al. (2015) studied the *α*-chymotrypsin-CNT system both in aqueous and heptane media. The results showed that, although the immobilization of the enzyme caused significant structure deviation from the native structure, insignificant changes in secondary structure were observed. Moreover, CNT was found to display a stabilization role in retaining the catalytic H-bond network, which was associated with the enhanced activity observed. Zhao and Zhou. (2017) studied the structure-function relationship of *α*-chymotrypsin (*α*-ChT) in interaction with pristine CNTs and carboxylated CNTs. It was observed that while interacting with the pristine CNTs through hydrophobic forces the active site of *α*-ChT is facing towards the solution and hence shows a non-competitive arrangement. However, the active pocket of the enzyme binds to carboxylated CNTs through a dominant electrostatic interaction which inhibits the enzyme in a competitive-like mode. In addition to this work, Di Giosia et al. (2020) studied the α-chymotrypsin-pristine CNT interactions using MD simulations together with spectroscopic, microscopic and kinetics experiments. They showed that CNT was occupying the α-chymotrypsin substrate binding site, reducing its available volume and therefore competing with the substrate and hampering the activity of the enzyme. The pristine and carboxyl-functionalized CNTs were further studied as immobilizing agents using the D-lactate dehydrogenase enzyme (Zaboli et al., 2019). It was observed that D-lactate dehydrogenase displayed an improved thermal stability when immobilized in comparison to the free enzyme. The simulations showed that the hydrogen bonding networks occurring between the enzyme and the functional groups of fCNT were responsible for maintaining the enzyme conformation more than was observed for pristine CNT. CNT surfaces were also used in order to understand the enzyme activation mechanism in non-aqueous media. The subtilisin carlsberg (SC) enzyme was immobilized onto CNT using water, acetonitrile and heptane as solvents (Zhang et al., 2016). It was found that the affinity of SC on CNT decreases with acetonitrile and in more degree with heptane, in contrast to water. This was explained by observing that the substrate binding pocket appeared significantly expanded by immobilization in the presence of acetonitrile and heptane. In a very recent work, Xu et al. (2022) studied the structural basis for the immobilization of a laccase enzyme on SWCNT. They used multi-scale simulations to gain insight into the direct electron transfer (DET) event of the *Thermus thermophilus* laccase (TtLac) adsorbed on carboxyl- and amino-functionalized CNTs (COOH-CIN and NH_2_-CNT respectively). It was observed that the laccase stability and its catalytic efficiency are more preserved when immobilized on NH_2_- than in COOH-CNT, since the enzyme undergoes less conformational perturbation. Other recent studies have focused on the interaction of CNTs with other biomolecules such as hormones and receptors (Mahmoodi et al., 2018; Zhang et al., 2018).

## 5 Immobilization on other surfaces

This section of the review covers structural studies of enzymes that have been immobilized on other surfaces such as polymers, membranes or zeolite. Petrosino et al. (2019) used a polysulfone (PSU) membrane surface to immobilize a phosphotriesterase (PTE) at a fixed pH. Hybrid quantum mechanics/molecular mechanics (QM/MM) was used to calculate the interaction energies between the enzyme and the surface, finding good agreement with the experimental adsorption free energies and supporting that PTE was effectively adsorbed on PSU. Interestingly, it was also observed that, with respect to the orientation observed onto pristine PTE, there was less accessibility for the immobilized enzyme binding site. This is because of the steric hindrance to the polymer, which may lead to a reduction in catalytic efficiency of the enzyme. In a recent work, the adsorption-desorption mechanisms of lysozyme over three antifouling polymer membranes were studied through MD simulations (Zhang et al., 2022). These are poly(3-(methacryloyloxy)propane-1-sulfonate) (T4-SP), poly(sulfobetaine methacrylate) (T4-SB), and poly(2-(dimethylamino)ethyl methacrylate) (T4-DM) which are grafted on polysiloxane membranes. The antifouling membranes lie in the core to prevent membrane fouling. The results showed that, after adsorption of lysozyme, the interaction is higher for T4-SP than T4-SB, being T4-DM showing the least interaction. The overall structure of the enzyme was not observed to change during the adsorption. However, slight fluctuation was noticed near the binding sites which was caused by structural adjustment for tighter combination. Although T4-DM has lowest interaction energy with lysozyme, the desorption of lysozyme on T4-DM is the hardest due to its larger hydrodynamic radius. The simulation results from this study are consistent with the experimental observation that T4-SB and T4-SP have good antifouling effects. Regarding other synthetic surfaces, Wei et al. (2011) studied the adsorption of lysozyme onto a polyethylene (PE) surface via MD simulations. The long axis of lysozyme, while adsorbed, was found to be parallel to the surface and displayed an anisotropic mobility over the surface. Interestingly, this observation is contrary to what was observed in the lysozyme adsorption to SNP (Hildebrand et al., 2015), but analogous to its adsorption on SAMs (Xie et al., 2013), as discussed in the previous sections. Additionally (Kubiak-Ossowska and Mulheran, 2010), also studied lysozyme adsorption on a model charged surface using MD simulations (Kubiak-Ossowska and Mulheran, 2010). The results showed that, although electrostatics steer the enzyme to a favorable binding orientation with respect to the surface, immobilization only occurs through the strong interaction of Arg128, a charged residue in a flexible location of the protein surface, with the model charge surface. More recently, the Zhou group combined MC and MD simulations to study the lysozyme adsorption on porous organic cages and MXenes (Zhao et al., 2020; 2021). For both surfaces van der Waals interactions-as well as electrostatics interactions for the later-played an important role in adsorption, while the conformation of the lysozyme remained stable suggesting a good biocompatibility. It was also observed that the interfacial water layer present over the surface plays a significant impact on adsorption. Regarding covalent attachment, an effective technique for irreversible enzyme immobilization, the cross-linking by glutaraldehyde (GA) is one of the most popular immobilization techniques. Hozhabr Araghi et al. (2021) combined MD simulations and quantum calculations to get a comprehensive molecular understanding of the interactions between the *β*-glucosidase (BGL) enzyme with propylamine-GA molecules. The results observed that all propylamine-GA molecules interacted with the lysine residues of BGL through their head side with Lys384, Lys376 and Lys247 being the most interactive residues. In another recent work Li et al. (2023) performed MD simulations along with surface residue microenvironment analysis to study thrombin adsorption on Ca^2+^ -exchanged LTA-type (CaA) zeolite. The observed thrombin deactivation on CaA zeolite seemed to be due to changes in the thrombin secondary structure upon adsorption. Additionally, some substrate binding sites of the enzyme are blocked by the zeolite surface after adsorption leading to the deactivation of thrombin. Moreover, few of the sites which bind heparin and fibrinogen are part of the positively charged area of thrombin which forms electrostatic interaction with CaA zeolite and plays an important role in thrombin adsorption. Since these sites are in vicinity to the catalytic sites of thrombin, this further affects thrombin coagulation activity on the CaA zeolite surface.

## 6 Immobilization on surfaces lacking molecular dynamics simulations

There are several surfaces on which immobilization of enzymes are studied experimentally but no detailed computational studies exist for them. For instance, due to several of their inherent properties, polymer brushes have received considerable attention as enzyme immobilization agents. Several studies have used brush polymers to immobilize lipase (Weltz et al., 2019; Weltz et al., 2020; Sánchez-Morán et al., 2021). A significant enhancement of the catalytic performance of *Bacillus subtilis* lipase A (lipA) was observed when immobilized on poly(sulfobetaine methacrylate) brushes, PSBMA. This was because of the stabilization of lipA structure through changes in its conformational dynamics, which resulted in the enhancement of its catalytic performance, which strongly depends on the chemistry of the brush. The basic mechanism for this structural stabilization by multipoint covalent immobilization to the brush polymer was also studied. The results showed that, with the increase in the number of lipA-brush attachments, the enzyme stability is increased and it is correlated directly with the enzyme rigidification. Additionally, several structurally diverse but related lipases were immobilized on random copolymer brush surfaces made up of sulfobetaine methacrylate (SBMA) and poly(ethylene glycol) methacrylate (PEGMA) aiming to shed light on the design of synthetic materials for enzyme stabilization. The results showed that the thermal stability of each lipase was strongly dependent on the fraction of PEGMA with respect to SBMA in the brush layer. However, a detailed structural understanding of this observation is lacking. The use of affinity tags or protein tags is another well established strategy used for enzyme immobilization (Ley et al., 2011). These are peptide sequences that are appended to proteins-normally at the N-terminus- so that they can be purified from their crude biological source. Polyhistidine tag, also known as His-tag, is one of the most commonly used affinity tags which is a string of usually between six to nine histidine residues. His-tag was used in recent works to immobilize β-glucosidase (Zhou et al., 2017), transketolase (Kulsharova et al., 2018), glucose dehydrogenase (Zhou et al., 2021), ketoreductase (Basso et al., 2022), and phosphomannose isomerase (Wang et al., 2023), between others. In a recent work, MD simulations were performed to study immobilization of fluorescent proteins through His-tag (Wasserberg et al., 2017). However, the application of this pipeline to predict sites for enzyme immobilization is still lacking. Metal-organic frameworks (MOFs) appeared recently as a promising immobilization material due to their attractive properties such as a high surface area, excellent stability, designable functionality, and tunable porosity (Ye et al., 2020). However, MOF-immobilization is still in its infancy and its mechanistic knowhow is still under development. Computational studies based on MD simulations may emerge as a tool to shed some light on these mechanisms and improve this arising immobilization strategy. Separately, recent studies have shown that magnetic nanoparticles (MNPs), which gained a special place as supporting matrices and versatile carriers, are considered a future trend for enzyme immobilization (Aber et al., 2016; Atacan et al., 2016; Mehrasbi et al., 2017; Zhou et al., 2017; Darwesh et al., 2019; Muley et al., 2020; Zanker et al., 2021; Zou et al., 2021). This is due to the easy recovery and reuse of MNPs by applying an external magnetic field in addition to their exceptional properties as nanoparticles like large surface-to-volume ratio, large surface area, high mass transfer and mobility (Bilal et al., 2018; Darwesh et al., 2020). However, molecular modeling studies in this field are still lacking and could shed light on the usage of MNPs, which paves the way towards an efficient green chemistry approach.

## 7 Computational protocols for predicting immobilization sites

The literature survey shown in above sections conveys that the computational studies were done primarily to rationalize the molecular aspects of the immobilization experiments. However, the use of state-of-the-art protocols that allow controlling the impact of immobilization on the enzyme properties is, up to date, very limited. In a recent work in this regard, the Knotts group tried to devise a reliable heuristics to identify optimal attachment locations in typical proteins (Smith et al., 2021), a protocol which may be promising when applied to enzyme immobilization. Using coarse-grained MD simulations they initially predicted how external factors-like confinement, tethering configuration, surface hydrophobicity and binding site valency-may affect protein stability and folding pathways (Rathore et al., 2006; Bush et al., 2015; Bush et al., 2017; Bush et al., 2018). Additionally, they performed tethering and PEGylation at a limited number of sites in the loop regions of lysozyme to prove the capability of predicting well-performing functionalization sites (Wei et al., 2010; Wei et al., 2011; Wei et al., 2013). Although no rigorous testing was performed for these heuristics, later they suggested regions involving secondary structures can be optimal functionalization sites (Wilding et al., 2018; Wilkerson et al., 2020). Very recently, they provided a screening protocol to search for accessible sites for functionalization on β-lactamase (TEM-1), either for tethering onto a surface or for PEGylation (Smith et al., 2021). The proposed heuristics of finding the accessible sites was also later validated through experiments (Soltani et al., 2022; Zhao et al., 2022). Overall, these studies may be a stepping stone to further design an adequate prediction protocol that helps to control and understand the structural and functional implications of the enzyme immobilization process, which will be key for its rapid implementation.

## 8 Conclusion

Immobilization of enzymes refers to physically confining or localizing enzymes in a defined region of space while preserving their catalytic performance. This phenomenon can significantly improve the stability, reusability, and enzyme lifetime. In addition to that, immobilization can also widen the enzyme application by further improving their stability under extreme conditions. Enzyme immobilization is often performed due to their use in applications like antimicrobial or/and antifouling coatings, drug delivery, industrial catalysis, biosensors or biofuel cells. Based on the different materials employed, various enzyme immobilization methods such as covalent bonding, adsorption, entrapment, and cross-linking were developed over the years. Although immobilization of enzymes has been a successful strategy to improve the catalytic properties of enzymes, in detailed understanding towards the correlation between the enzyme attachment site, the chemical properties of the attached surface, and activity of the enzyme remains elusive.

Over the past two decades, *in silico* investigations-in particular MD simulations-have emerged as an important tool to study the immobilization of enzymes that can disclose the microscopic nature of intermolecular interactions. Both all-atoms and coarse-grained MD simulations have been extensively used to study mechanistic details of enzyme immobilization on different surfaces like nanoparticles, SAM, graphene, CNT and others (summary presented in Table 1), often in combination with MC based approaches to study adsorption, the initial step of immobilization. These studies have revealed that enzyme performance can be tuned by controlling their orientation on a charged surface by the electric dipole. In the same manner, an hydrophobic dipole of the enzyme could be used as a criterion to predict its orientation on hydrophobic surfaces. Additionally, apart from the nature of the surface, the electrostatic potential distribution on the enzyme surface is also responsible for its orientation on the immobilizing material. Simulation studies of tethered enzymes show that tethering to multiple sites increases thermal stability of the enzyme. In addition, as speculated, they also reveal that immobilizing enzymes through positions that are far away from the active site leads to less disturbances in the catalytic regions, as well as that the choice of the solvent can also affect the enzyme immobilization, facts which are in accordance with the experimental observations.

**TABLE 1:** Summary of Molecular Dynamics simulation studies of immobilization of different enzymes on different surfaces.

Nanoparticles				
Enzyme	Material	Simulation method	Experiment	References
Papain	SNPs	MD, MM-PBSA	SDS-PAGE	He et al. (2014)
Cytochrome C	SNPs	MD	None	Sun et al. (2014b)
RNase A	SNPs	MD	None	Sun et al. (2014b)
Lysozyme	SNPs	MD	None	Sun et al. (2014b)
	SNPs	MD	Adsorption	Hildebrand et al. (2015)
	SNPs	CG-MD	None	Yu and Zhou (2016)
	Charged silica pore	CG-CpHMD	None	Caetano et al. (2021)
***α***-chymotrypsin	SNPs	MD	Adsorption	Hildebrand et al. (2015)
Lipase	SNPs	MD	FTIR-ATR	Wang et al. (2020)
Catechol O-methyltransferase	AgNPs	MD	SDS-PAGE, Adsorption, Fluorescence, FTIR	Usman et al. (2021)
Alcohol dehydrogenase	PPY-Tip nanocomposite	MD	FTIR, FE-SEM, TGA	Baig et al. (2015)
Tripsine	AuNPs	CG-MD	None	Tavanti et al. (2019)

Self Assembled Monolayer (SAM)				
Enzyme	Material	Simulation method	Experiment	References
Cytochrome C	CH_3_-&SH-SAMs	MD	None	Tobias et al. (1996)
	Organic SAM	MD	None	Nordgren et al. (2002)
	COOH-SAM	MC + MD	None	Zhou et al. (2004)
	Mercaptoundec-anoic acid SAM	MD	None	Rivas et al. (2005)
	PC-SAMs	MC + MD	None	Xie et al. (2015)
	NH_2_-SAM	MD	None	Peng et al. (2016)
Lysozyme	COOH-SAM	MC + MD	None	Xie et al. (2013)
	Mixed & methyl terminated SAMs	MC + MD	None	Zheng et al. (2004)
	OEG-,OH-& CH_3_-SAMs	MC + MD	None	Zheng et al. (2005)
	PC-SAM	MC + MD	None	He et al. (2008)
	COOH-&OH-SAMs	MC + MD	None	Xie et al. (2020)
Feruloyl esterase	COOH-&NH_2_-SAMs	MC + MD	None	Liu et al. (2015a)
Laccase	COOH-&NH_2_-SAMs	MC + MD	None	Liu et al. (2015b)
	4-ATP SAM	MD, MM-PBSA	None	Miyazawa et al. (2017)
RNase A	COOH-&NH_2_-SAMs	MC + MD, CG-MD	None	Liu et al. (2017)
	COOH-&NH_2_-SAMs and bare AuNPs	CG-BD	None	Bourassin et al. (2022)
Bilirubin oxidase	COOH-&NH_2_-SAMs	MC + MD	None	Yang et al. (2018)
Lipase	COOH-&NH_2_-SAMs	MC + MD	None	Zhao et al. (2015)
β-galactosidase	Maleimide/hydroxyl SAMs	CG-MD	SFG spectroscopy	Li et al. (2018)
Nitro-reductase	Maleimide/hydroxyl SAMs	CG-MD	SFG spectroscopy	Zou et al. (2018)
	COOH-&NH_2_-SAMs and bare AuNP	CG-BD	None	Bourassin et al. (2022)
Hydrogenase	SH-SAM	MD	SEIRA, AFM, PFV	Heidary et al. (2015)
Bilirubin oxidase	COOH-& NH_2_- SAMs and bare AuNPs	CG-BD	None	Bourassin et al. (2022)
*Torpedo californica*	COOH-& NH_2_-SAMs	MC + MD	None	Yang et al. (2020)

Graphene and Carbon nanotube (CNT)				
Enzyme	Material	Simulation method	Experiment	References
***α***-chymotrypsin	Graphene & GO	MD	None	Sun et al. (2014a)
	CNT	MD	None	Zhang et al. (2015)
	Pristine & carboxylated CNT	MC + MD	None	Zhao and Zhou (2017)
	Pristine SWCNT	MD, MM-GBSA	None	Di Giosia et al. (2020)
Cytochrome C	Graphene & GO	MD	None	Zhao et al. (2018)
Lipase	GO	MD	AFM, UV, CD	Mathesh et al. (2016)
	GO	MD	SEM, TEM, TGA, MS, XPS, XRD	Zhuang et al. (2020)
D-psicose 3-epimerase	GO	MD	None	Li et al. (2021)
Lysozyme	GO	MD, MM-GBSA	UV-Vis, ITC, Fluorescence, CD, DSC	Bera et al. (2018)
	SWCNT	CG-MD, REMD	None	Vaitheeswaran and Garcia (2011)
	CNT	MD, MM-PBSA	None	Calvaresi et al. (2012)
Nucleoside diphosphate kinase	GO	MD	None	Ray et al. (2018)
Coxsackie-Adenovirus Receptor	CNT	MD	None	Johnson et al. (2009)
Human serum proteins	SWCNT	MD	AFM, Fluorescence spectroscopy, CD, SDS-PAGE	Ge et al. (2011)
D-lactate dehydrogenase	Pristine and carboxylated CNT	MD	Fluorescence spectroscopy	Zaboli et al. (2019)
Subtilisin Carlsberg	CNT	MD	None	Zhang et al. (2016)
Laccase	SWCNT	MC + MD	None	Xu et al. (2022)

Others				
Enzyme	Material	Simulation method	Experiment	References
Phosphotriesterase	PSU	QM/MM	None	Petrosino et al. (2019)
Lysozyme	Antifouling membranes	MD	None	Zhang et al. (2022)
	Polyethylene	MD	None	Wei et al. (2011)
	Porous organic cage	MC + MD	None	Zhao et al. (2020)
	Mxenes	MC + MD	None	Zhao et al. (2021)
β-glucosidase	Propylamine-GA	MD, quantum calculations	None	Hozhabr Araghi et al. (2021)
Thrombin	Zeolite	MD	None	Li et al. (2023)

In spite of tremendous efforts made on investigating the enzyme immobilization phenomenon through *in silico* simulations, several key issues still require further exploration. The fact that a large variety of immobilizing agents exists, makes it difficult to decide which is the most adequate for a particular enzyme, and *in silico* strategies helping in this direction could speed up this selection process. Moreover, the simulations performed till date in this regard were primarily to rationalize the molecular aspects of the immobilization experiments. Only a very limited effort has been made in the use of computation to predict adequate protocols that can control the impact on the enzyme properties and the research field is still in its infancy. Overall, state-of-the-art force-field based computational methods like MD simulations are tedious and might not be the best approach to meet the industrial needs for predicting immobilization sites. In contrast, data driven methods like Machine Learning (ML)-based approaches might be key in the near future to design better pipelines that can aid in the growing immobilization field. For the time being, developing pipelines based on distinct levels of accuracy allowing a proper sampling and determination of the enzymatic performance might profoundly minimize the time consumption, costly trials, and investigation errors when developing highly efficient immobilized enzymes.
